# *Codonoboea personatiflora* (Gesneriaceae), a new species from Peninsular Malaysia

**DOI:** 10.3897/phytokeys.18.3487

**Published:** 2012-12-07

**Authors:** Ruth Kiew, Yen-Yen Sam

**Affiliations:** 1 Forest Research Institute Malaysia, 52109 Kepong, Selangor, Malaysia

**Keywords:** *Codonoboea personatiflora*, Gesneriaceae, new species, Terengganu, Peninsular Malaysia, personate flower

## Abstract

*Codonoboea personatiflora* Kiew & Y.Y.Sam, **sp. nov.**, is described from lowland forest in the foothills in Terengganu, Peninsular Malaysia. It is unique in the genus in its personate flower. Its conservation status falls within the IUCN Endangered category.

## Introduction

This striking new species has attracted attention for a number of years for its tall bushy habit and glossy young leaves conspicuously reddish towards the base and bright yellowish green towards the apex. However, it was only when it was cultivated in the nursery of the Forest Research Institute Malaysia in Kuala Lumpur that flowering material could be obtained and the species described. It belongs to *Codonoboea*, a genus of about 120 species that is an important component of the herb layer in rain forest in W Malesia extending as far north as Southern Thailand and as far south as Sulawesi (Kiew and Lim 2011). Its centre of distribution with 79 species is Peninsular Malaysia where it is found in abundance and diversity (Kiew 2009). Recent molecular phylogenetic analyses (Weber et al. 2011) have confirmed that *Codonoboea* is monophyletic and is a genus distinct from *Didymocarpus* (Weber and Burtt 1983), *Henckelia* and *Loxocarpus* (Weber and Burtt 1998). It is unique among *Codonoboea* species in its personate (closed) flower.

## Taxonomy

### Codonoboea personatiflora

Kiew & Y.Y.Sam sp. nov.

urn:lsid:ipni.org:names:77123717-1

http://species-id.net/wiki/Codonoboea_personatiflora

Figure 1 Map 1

#### Diagnosis.

Different from all other species of *Codonoboea* Ridl. in its personate flower where the lower lip is directed upwards and completely closes the mouth of the corolla tube.

#### Type.

**Peninsular Malaysia.** Terengganu: Kemaman, Sungai Nipah, Bukit Kajang, 4°20'N, 103°07'E, 22 Nov 1935, E.J.H.Corner SFN 30540 (holotype: SING!; isotypes: K!, L, E, SAR!).

Robust, erect, unbranched herb. **Stem** woody to 65 cm tall, flowering at ca 15 cm tall, bare stem below the leaves 4–9 mm diam. **Leaves** opposite, pairs equal, spaced 8–17 mm apart, glossy when young, reddish towards the base and bright yellowish green toward apex, older leaves uniformly dull greyish green above; petioles 1.3–3 cm long, slightly grooved above, minutely pubescent; lamina oblanceolate, thinly leathery, 15–34 × 4–8 cm, lateral veins whitish or yellowish green, sometimes with a silvery band along the midrib, paler beneath, base narrowed then slightly rounded, margin serrate in the upper half, teeth ca 1 mm long, apex narrowly acuminate, acumen to 1–2.5(–4) cm long; midrib and veins impressed above, prominent beneath in life, lateral veins 9–20 pairs, ferrugineous beneath.

***Inflorescence*** a pair-flowered dichasial cyme with up to 5^th^ order branching, erect from the upper leaf axils, 12–34 cm long, peduncle purple or reddish brown, 7.5–29 cm long, bracts green, linear, 2–10 × ca 0.5 mm, pedicels 2–5 mm long. Inflorescence, pedicel and calyx with minute, dense, non-glandular pubescence. **Flowers** pendent, to 20 mm long, almost hanging vertically down, buds green or only at the base; calyx brownish purple or green, ca 1.5–2 × 0.75 mm long, 5-lobed divided almost to the base, apex acute; corolla pale cream with pale pink lobes, personate, densely glandular pubescent outside, tube 9–13 × 3–5 mm, lower half of tube cylindric, the upper half gradually dilating to ca twice the width of the basal half, mouth 6–9 mm diam., upper lip erect, glabrous, ca 7 mm long with 2 strongly reflexed lobes, each ca 2.5 × 2.5 mm with a raised patch of long glandular hairs between lobes, lower lip positioned upwards and appressed against the upper lip closing the corolla mouth, 8–10 × 6.5–9 mm long, the 3 rounded, glabrous lobes reflexed to cucullate, raised and forming a rim, lateral lobes ca 2 × 2 mm, central lobe ca 3 × 2.5 mm, throat with dense patch of deep pink unbranched long multiseriate glandular hairs, nectar guides 2, strongly raised, concolorous with the tube; stamens 2, joined 4–5 mm from corolla base, filaments white, slightly bowed inwards, 7–13 mm long with 2 blunt horns at the attachment to the anther, anthers purple or pale cream, ca 2 × 0.75 mm, cohering face-to-face, staminodes 2, slender ca 3 mm long, hooked at apex; nectary cylindric, margin unequally lobed, upper lobes 2–3 mm long, lower lobes 2–3.5 mm long; ovary and style slender, with dense brownish glandular hairs, ovary white, 5–8 × 0.75–1 mm long, style purple-brown, 5–6 mm long, stigma globose, almost 0.5 mm diam. The anthers and stigma are positioned at the same level just below the upper lip. **Fruits** slender, cylindric, 22–45 × 1–1.5 mm, dehiscing along the upper suture.

**Figure 1.**
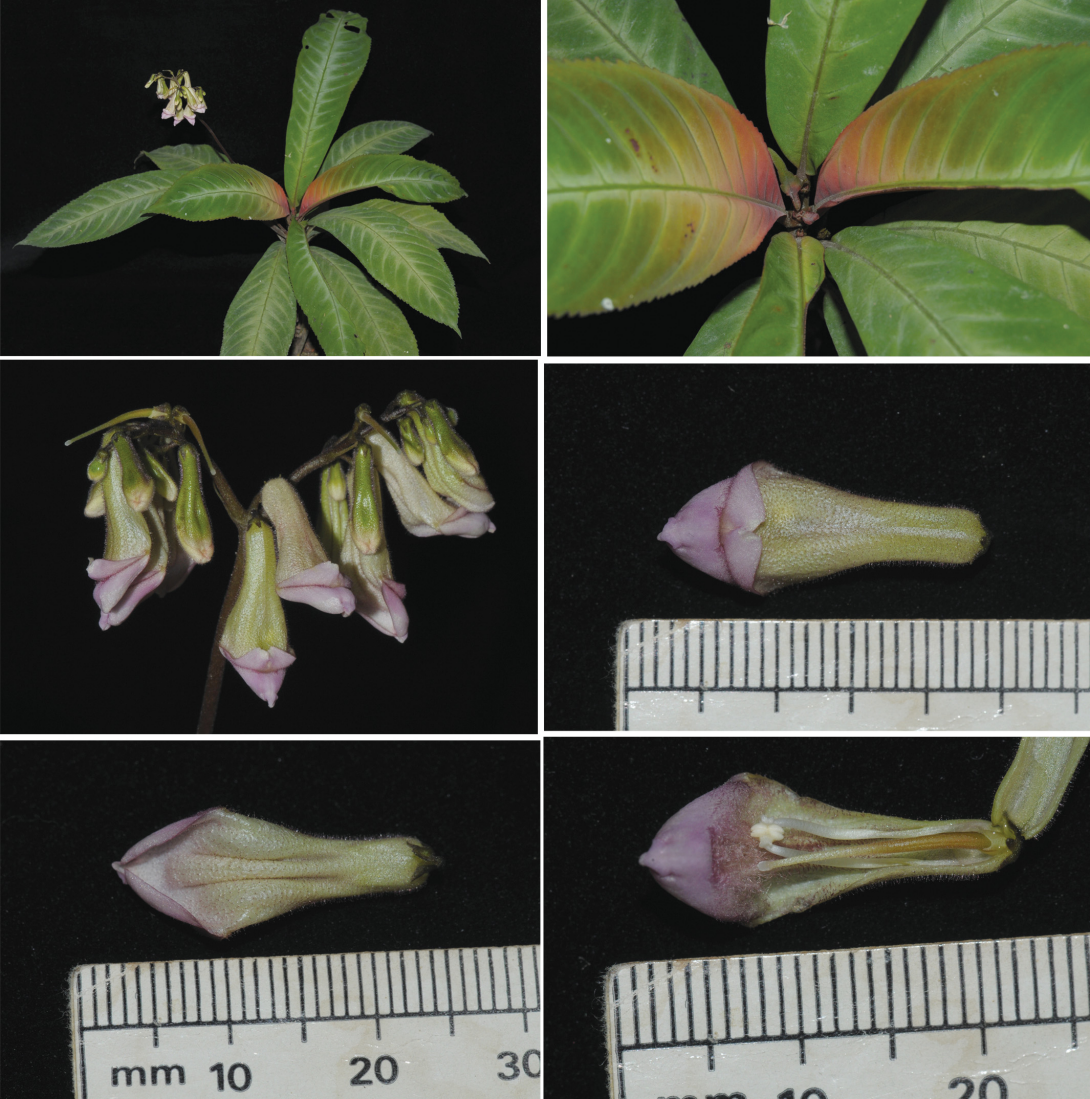
*Codonoboea personatiflora* Kiew & Y.Y.Sam. **A** habit **B** coloured young leaves **C** pair-flowered cymose inflorescences **D** flower (top view) **E** flower (from below) **G** longitudinal section with upper lip removed.

#### Distribution.

Peninsular Malaysia, endemic in the state of Terengganu.

**Map 1.**
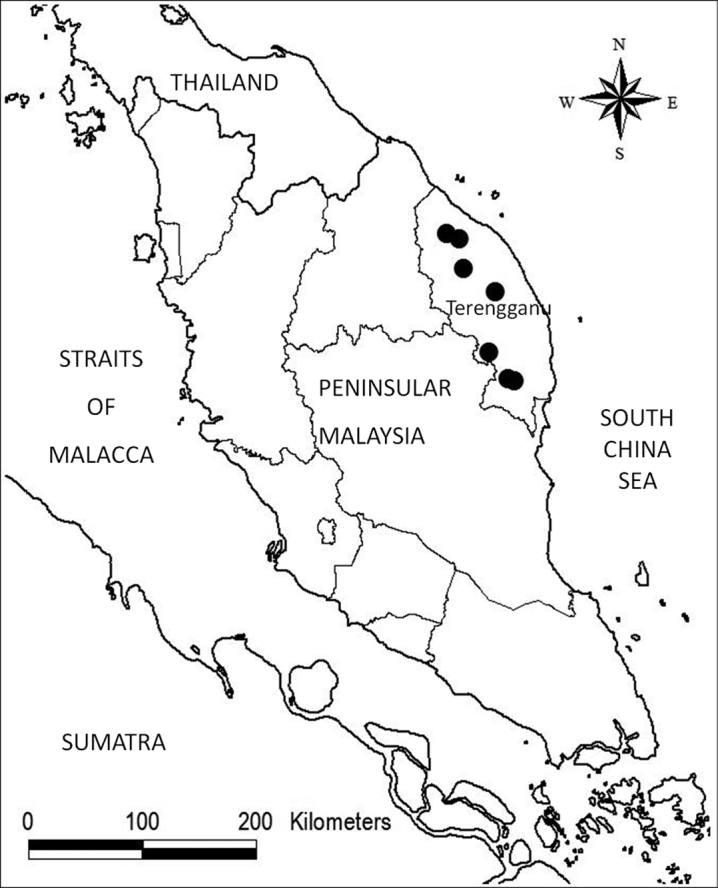
Distribution of *Codonoboea personatiflora* in Peninsular Malaysia.

#### Ecology.

In primary or logged-over lowland mixed dipterocarp forest at low altitudes (below 100 m), on shaded hillsides or slopes, often above streams.

#### Etymology.

Named for its personate (closed) flower that is unique for the genus.

#### Conservation status.

EN B2a,b(ii,iii). Following the IUCN Criteria and Categories (IUCN 2001; Chua 2010), this species falls within the Endangered Category because it is known from five localities, has in total an area of occupancy of 28 sq km. In addition, it is nowhere found within Peninsular Malaysia’s network of Totally Protected Areas and it grows at low altitudes in lowland forest, a habitat that is threatened by or already has been logged or is threatened by forest clearance for oil palm plantations.

#### Other specimens examined.

**Peninsular Malaysia**. Terengganu: Dungun, Jengai Forest Reserve, Compartment 71, 15 Mar 1998, Anon. s.n. (SING!), Compartment 52, 65 m alt., 4°32.76'N, 102°58.34'E, 17 Oct 2002, Y.Y.Sam FRI 47153 (KEP!, SAN!); Hulu Terengganu, Ulu Telemong Forest Reserve, 5°12'N, 102°46'E, 25 Jul 2006, R.Kiew RK 5339 (KEP!, K!, SAR!); Hulu Terengganu, Ladang Ternakan TERSAT, 62 m alt., 5°01.07'N, 103°01.17'E, 31 Oct 2009, M.Kamarul-Hisham FRI 67168 (KEP! SING!); Kemaman, Sungai Nipah Forest Reserve, Jeram Tanduk, 81 m alt., 4°18.93'N, 103°10.42'E, Y.Y.Sam FRI 47197 (KEP! SING!); probably from Kemaman, Sungai Nipah, collector unknown, flowered in Forest Research Institute Malaysia Nursery on 9 Apr 2012, FRI 75314(KEP!); Setiu, Ulu Setiu Forest Reserve, 5°26'N, 102°44'E, 7 Mar 2002, Y.Y.Sam FRI 44395 (KEP!), flowered in Forest Research Institute Malaysia Nursery on 9 Sept 2003, Y.Y.Sam FRI 44483 (KEP!).

#### Discussion.

This new species belongs to *Codonoboea* sect. *Didymanthus* in its erect habit, petiolate leaves in distant pairs, cymose inflorescences with a long peduncle and inconspicuous bracts, and medium-sized (to 2 cm long) flowers (Ridley 1923). However, it differs from the other species in this section, and indeed from all other known *Codonoboea* species, in its personate flower. The special feature of this flower is the lower lip where the recurved lobes form a rim that is pressed upwards against the upper lip and so closes the corolla mouth, unlike the usual open funnel- or trumpet-shaped or campanulate corolla of *Codonoboea* species. In addition, the aperture is filled by the long hairs, a feature not seen in other *Codonoboea* species. The lower lip appears to be hinged because it is readily bends downward when pressure is applied so opening up the mouth of the corolla tube.

The majority of *Codonoboea* species are nectar flowers (the cylindrical nectary surrounding the base of the ovary supplies nectar as the reward for the pollinator) with a narrow tubular flower that is strongly dilated to produce an open funnel- or trumpet-shaped flower, often with the lower lobes projecting and forming a landing platform for the insect pollinator. Usually there are yellow or orange nectar guides on the floor of the tube. The flower is therefore open to any insect small enough to enter the tube. Although there are very few observations on pollination in this genus, bumblebees (*Bombus* sp.) have been observed visiting two species, *Codonoboea hispida* (Ridl.) Kiewand *Codonoboea robinsonii* (Ridl.) Kiew, both belonging to *Codonoboea* sect. *Didymanthus* (Kiew 2009) and it is likely, based on floral morphology, that bees are the pollinators of most of these *Codonoboea* species. The other type of flower seen in the genus is the smaller, short-tubed, often campanulate pollen flower where the anthers are large and conspicuous in the mouth of the corolla. These flowers either have very small nectaries or none at all and pollen is offered as the reward.

This new species is obviously a nectar flower but is unique in *Codonoboea* in that the upper and lower lips fit closely together so that the mouth is closed and in addition there is a tangle of long hairs just inside the mouth that might prevent small insects from squeezing between the lips. This personate flower closely resembles that of the ornamental snapdragon, *Antirrhinum majus* L. (Scrophulariaceae) that is pollinated by bumblebees that on landing on the flower are sufficiently heavy to depress and open the lower lip.

There are only two other examples of this type of personate flower in Malaysian Gesneriaceae, namely in *Didymocarpus antirrhinoides* A.Weber (Weber and Burtt 1983) that has a flower of similar size (15–23 mm long) and *Rhyncholossum medusothrix* B.L.Burtt (Burtt 1962) with a corolla tube 10–25 mm long that in addition has ‘medusoid’ hairs in the throat. However, for none of these personate flowers is the pollinator known.

## Supplementary Material

XML Treatment for Codonoboea personatiflora
